# Analysis of Osteoclasts and Root Resorption in Corticotomy-Facilitated Orthodontics with Ibuprofen Administration—An Animal Study

**DOI:** 10.3390/dj10090170

**Published:** 2022-09-08

**Authors:** Chanakant Jindarojanakul, Pannapat Chanmanee, Bancha Samruajbenjakun

**Affiliations:** 1 Orthodontic Section, Department of Preventive Dentistry, Faculty of Dentistry, Western University, Pathum Thani 12150, Thailand; 2 Orthodontic Section, Department of Preventive Dentistry, Faculty of Dentistry, Prince of Songkla University, Songkhla 90110, Thailand

**Keywords:** orthodontics, corticotomy, ibuprofen, osteoclasts, root resorption

## Abstract

Following corticotomy surgery, patients experience moderate to severe post-operative pain that necessitates prescriptions of analgesics. The prostaglandin inhibitory effect of ibuprofen influences the mobility of teeth during orthodontic treatment. This study aimed to determine how ibuprofen affects histological reactions and dental root resorption during orthodontic tooth movement aided by corticotomy. Forty-two male Wistar rats were divided into three groups by random selection: (1) control group, (2) corticotomy group (CO), and (3) corticotomy with 0.6 mL of 15 mg/kg ibuprofen group (CI). On each buccal and palatal alveolar bone, two decortication points were made. Orthodontic tooth movement was induced on the maxillary first molar for 21 days utilizing a NiTi-closed coil spring with 10 g of force. Hematoxylin and eosin were used to prepare and stain the histological sections. The numbers of osteoclasts on days 0, 7, 14, and 21 were determined, and the root resorption area on days 0 and 21 was measured. Compared to the control group, the osteoclast counts in the CO and CI groups were considerably greater (*p* < 0.002). No significant differences were observed between the CO and CI groups in the numbers of osteoclasts or the percentages of root resorption (*p* > 0.05). The amounts of osteoclast activity and root resorption were unaffected by the administration of ibuprofen in corticotomy-facilitated tooth movement.

## 1. Introduction

More attention is being placed on speeding tooth movement procedures in orthodontic research to shorten the length of treatment times. The numerous negative effects of prolonged treatment include an increased risk of gingival irritation, tooth decalcification, caries, and root resorption [1]. A statistically significant association between root resorption and treatment duration was discovered in earlier investigations on longer orthodontic treatment times [2]. Several methods that recommend speeding tooth movement with the least disadvantages include administration of exogenous biological molecules, vibration, low-level laser therapy, and surgical approach [3].

Orthodontic treatment accelerated by corticotomy is a therapeutic, surgical procedure that improves bone metabolism and speeds tooth movement [4]. The regional acceleratory phenomenon, which is a local reaction of tissues to noxious stimuli that stimulate the tissue to regenerate more quickly than normal [5], is the foundation of this technique. Corticotomy-facilitated orthodontic tooth movement potentially accelerates tooth movement by a magnitude of two to four compared to conventional orthodontic treatment [6,7]. The procedure is harmless and has no significant effect on root resorption [6]. Therefore, corticotomy is effective in accelerating tooth movement, resulting in a reduced treatment time [8].

In a study by Al-naoum et al. [7], a pain evaluation revealed that 20% of patients experienced severe pain throughout the day following corticotomy surgery, and 50% of patients experienced acute pain when eating. After a week, 33% and 47% of the patients continued to report moderate pain during the day and between meals, respectively. Analgesics are a post-operative pain treatment strategy for patients to reduce discomfort.

Ibuprofen is a potential non-steroidal anti-inflammatory drug used to treat moderate to severe dental discomfort [9,10]. This drug works by inhibiting the cyclooxygenase activity at the site of tissue injury to suppress prostanoids and prostaglandin E2 (PGE_2_), which is the main mediator in the pain signaling process [11]. The administration of ibuprofen, along with orthodontic tooth movement, was found to lower PGE_2_ levels [12,13] and influence osteoclastogenesis by reducing the number of osteoclasts [13] as a result of decreasing tooth movement [13,14,15]. In the rat model, the use of ibuprofen was found to have a significant effect on reduced root resorption [15].

The corticotomy-accelerated orthodontic tooth movement with or without ibuprofen intake exhibited no statistically significant differences in tooth movement or bone volume fraction [16]. The consequences on the response of the alveolar bone and the root have not been fully investigated. The aim of this study was to investigate the effects of ibuprofen administration on the number of osteoclasts and the degree of root resorption induced by corticotomy-facilitated orthodontic tooth movement.

## 2. Materials and Methods

**Figure 1:**
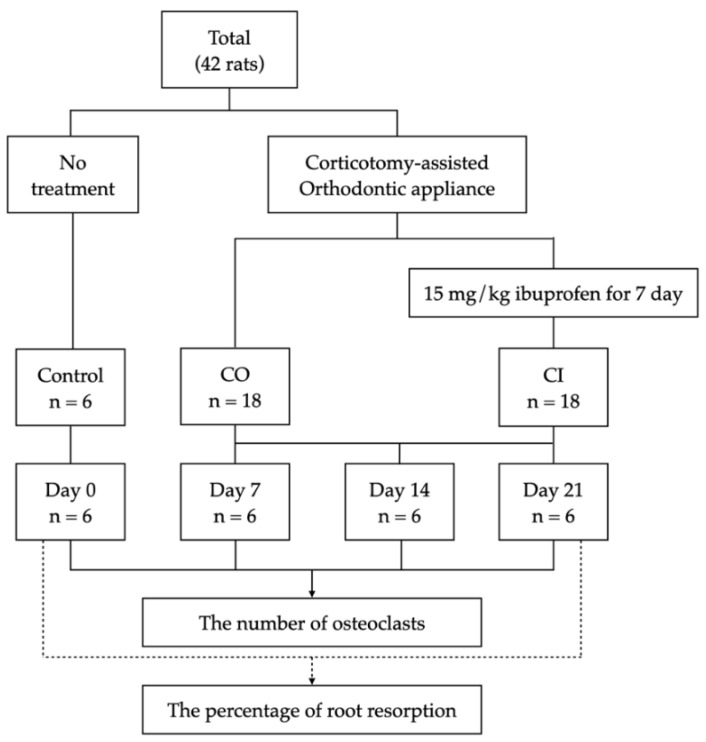
Schematic representation of the experimental design and timeline.

This study was approved by the Animal Ethics Committee, Southern Laboratory Animal Facility, Faculty of Science, Prince of Songkla University (IACUC 2563-03-037). Forty-two male Wistar rats (Rattus norvegicus albinos) aged 3 to 4 months and weighing 350 to 400 g were used for the experiments. Prior to the experiment, all animals were raised and housed in a sterilized room with a photoperiod of 12/12 h beginning at 7 a.m., a controlled temperature of 25 °C, and humidity of 40 to 50%. The animals were randomly categorized into three groups: (1) control group (control), which received no treatment for a baseline on day 0 (*n* = 6); (2) corticotomy group (CO), which received only corticotomy-assisted orthodontic tooth movement (*n* = 18); and (3) corticotomy-assisted orthodontic tooth movement with ibuprofen intake group (CI) (*n* = 18) (Figure 1).

The rats underwent corticotomy and orthodontic appliance insertion operations under general anesthesia with anesthetic doses adjusted to the weight of each rat. A combination of 90 mg/kg of ketamine hydrochloride (Ketajex™, Baxter Pharmaceuticals India Private Limited, Ahmedabad, Gujarat, India) and 10 mg/kg of xylazine hydrochloride (X-Lazine, L.B.S. Laboratory Ltd., Bangkok, Thailand) was intraperitoneally injected to induce anesthesia after inhalation of 3% isoflurane (Aerrane, Baxter Healthcare Corporation, Deerfield, IL, USA).

### 2.1. Alveolar Corticotomy Procedure 

**Figure 2:**
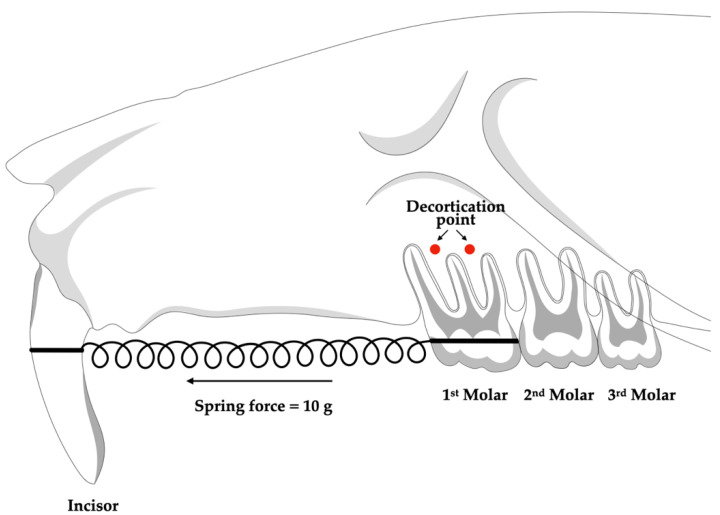
Schematic drawing of corticotomy points and the orthodontic application placement.

Each animal in the CO and CI groups had one side of its maxilla randomly chosen to carry out the corticotomy-assisted orthodontic tooth movement. The full-thickness flaps on the buccal and palatal sides were elevated after a sulcular incision with a number 11 blade in the gingival sulcus of the maxillary first molar to perform a corticotomy. By applying a 0.5 mm diameter carbide round bur with a slow-speed handpiece and sterile water irrigation, two decortication sites measuring 0.25 mm in depth and 0.5 mm in width were generated on the buccal and palatal sides of each tooth [17]. An absorbable suture (Novasorb, Novamedic Co., Ltd., Samut Prakan, Thailand) was used to seal the flap (Figure 2).

### 2.2. Orthodontic Application Placement

An ultra-light nickel–titanium closed coil spring (Dentos, Daegu, Korea) with dimensions of 8 mm in length and 1.5 mm in diameter was used to apply a 10 g force between the maxillary first molar and both maxillary central incisors to induce mesial orthodontic tooth movement in the maxillary first molars. At the cervical margin, a 0.5 mm deep retention groove was made on the labial and distal surfaces of both central incisors. In order to prevent the spring from breaking, both sides of the closed coil were ligated with 0.008″ ligature wire (Unitek™ Ligature Wire, 3M ESPE, Saint Paul, MN, USA) and covered with light-cured flowable composite resin (Filtek™ Z350 XT, 3M ESPE, Saint Paul, MN, USA). Every seven days, the force was reassessed, and the closed coil spring was reactivated or rebounded as necessary (Figure 2).

### 2.3. Administration of Medication

Solutions were administrated as a single oral dose via a gastric tube every day for seven days. The CI group received 0.6 Ml of 15 mg/kg oral suspension of ibuprofen (Nurofen syrup, Reckitt Benckiser, Samut Prakan, Thailand), while the CO group received 0.6 Ml of reverse osmosis filtered water [18].

### 2.4. Histological Approach

**Figure 3:**
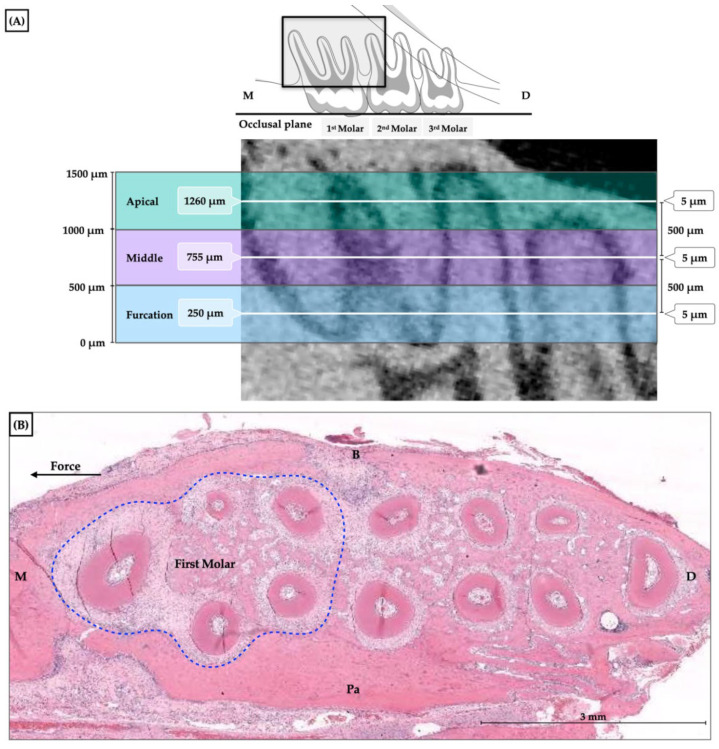
The histological sections. (**A**) The three levels of roots: cervical, middle, and apical levels with 5 µm in thickness at 500 µm intervals; (**B**) The hematoxylin and eosin-stained histological sections. M = mesial, D = distal, B = buccal, P = palatal.

Six animals in each group were euthanatized with a high dose of the anesthetic drug on days 0 (baseline), 7, 14, and 21 of orthodontic tooth movement, and the maxilla was removed. The maxilla was fixed in 10% formalin for 7 days and decalcified with 10% ethylenediaminetetraacetic acid (EDTA) at Ph 7 for 21 days at room temperature followed by dehydration with graded alcohols. Horizontal preparations of paraffin-embedded serial sections of the maxilla parallel to the occlusal plane of the maxillary first molar were then conducted. Each specimen was divided into three levels: cervical, middle, and apical levels. The section started at 250 μm underneath the furcation region. Each section had a thickness of 5 μm with a 500-micrometer interval. Hematoxylin and eosin were used to stain the sections to observe the general features of bone remodeling (Figure 3).

### 2.5. Measurement of Osteoclast Numbers

**Figure 4:**
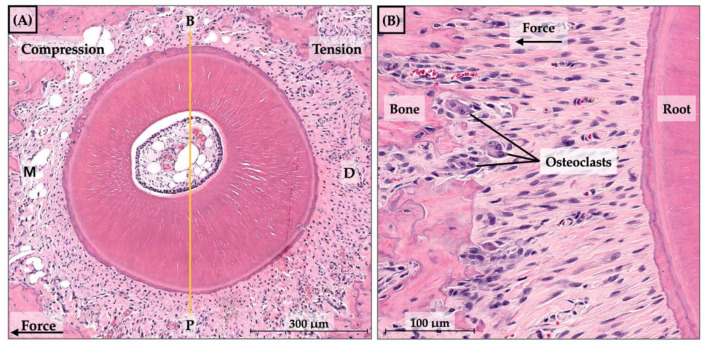
(**A**) Histological sections of the distopalatal root of the maxillary first molar (magnification, 40×), M = mesial, D = distal, B = buccal, P = palatal. (**B**) Details of osteoclast cells around the alveolar bone surface.

The number of osteoclasts was counted in the specimens collected at baseline, days 7, 14, and 21. The maxillary first molar distobuccal and distopalatal roots were used in the analysis, and the buccopalatal axis was used to divide the roots into compression and tension sides. The quantification of osteoclasts was performed using a captured picture at 40× magnification from a bright field microscope with the Aperio slide scanner and image viewing software version 12.3.2 (Aperio CS2 Digital Pathology Slide Scanner and Aperio ImageScope-Pathology Slide Viewing Software, Leica Biosystems Imaging Inc., Deer Park, IL, USA). The number of osteoclasts was counted on the compression side using a histological criterion that categorizes them as multinucleated cells with the presence of two or more nuclei (Figure 4).

### 2.6. Measurement of Root Resorption

**Figure 5:**
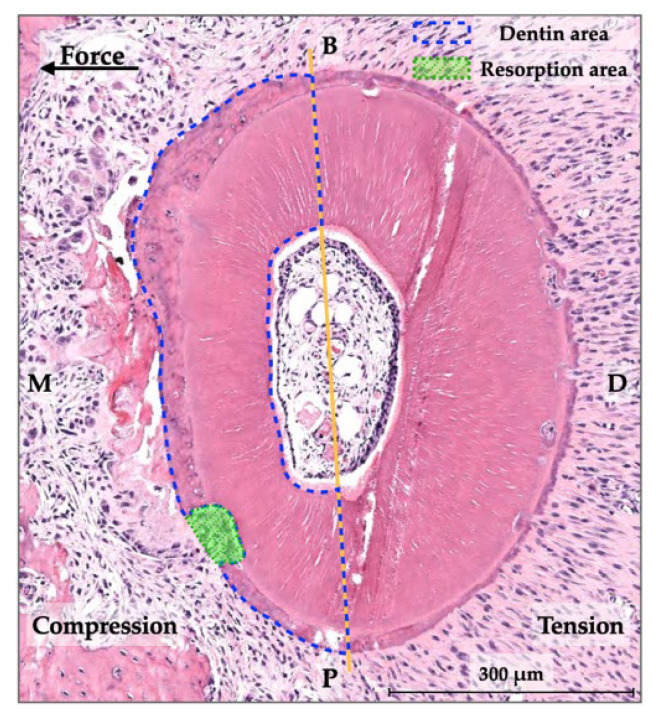
Histomorphometric analysis of root resorption. M = mesial; D = distal; B = buccal; P = palatal (magnification, 40×).

Root resorption of the distobuccal and distopalatal roots in the mesial half (compression side) of the maxillary first molar was assessed by histomorphometric analysis using samples from baseline and day 21. The root resorption area was defined as the enclosed area of the resorptive root surface and the imaginary line that connected the margins of each discontinuity of the root surface. In order to calculate the proportion of root resorption, root resorption areas on the compression side (mesial side) were compared to the combination of root resorption areas and dentin areas on the mesial side (Figure 5) [17]. The root resorption area and dentin area were measured using image viewing software (Aperio). After determination of the mean and standard deviation values of the root resorption percentages from the three sections (cervical, middle, and apical levels), the root resorption percentages in the control group, CO group, and CI group were compared.

### 2.7. Statistical Analysis

All samples were measured twice at one-month intervals, and intraobserver reliability was assessed using intraclass correlation coefficients. SPSS version 28 (IBM Corp, Armonk, NY, USA) was used for the statistical analyses. The Shapiro–Wilk tests indicated that the data were normally distributed. The number of osteoclasts on each day and the percentage of root resorption results were analyzed by one-way analysis of variance (ANOVA). A *p*-value less than 0.05 was considered to be statistically significant.

## 3. Results

All orthodontic appliances remained in place until the end of the experiment. The intraclass correlation coefficients for the number of osteoclasts were 0.977 in the distobuccal root and 0.865 in the distopalatal root, and the histomorphometric analysis of root resorption was 0.953 and 0.931 in the distobuccal and distopalatal roots, respectively, which indicated good to excellent reliability [19].

### 3.1. Numbers of Osteoclasts

In all time periods, the numbers of osteoclasts in the CO and CI groups were greater than four times the control group, which was statistically significant (*p* < 0.002). In both the CO and CI groups, the number of osteoclasts increased to a peak during the first week of treatment and subsequently gradually decreased from the second to the third week. Although the CO and CI groups noticed a similar pattern of changes in osteoclast counts, the CI group consistently had an insignificantly lower number of osteoclasts than the CO group throughout each trial period.

**Figure 6:**
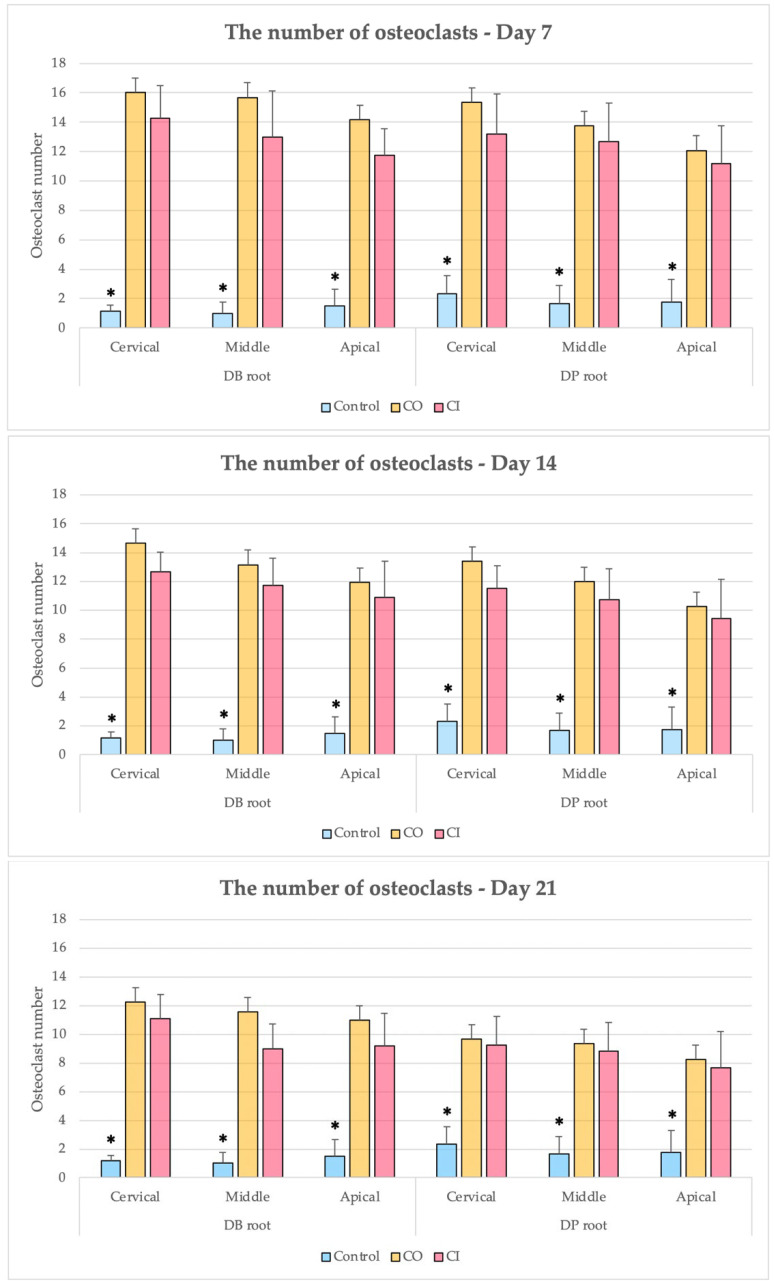
Comparisons between control, CO, and CI groups on days 7, 14, and 21. * *p* < 0.05.

The numbers of osteoclasts at the cervical level of the CO and CI groups were slightly greater than at the middle and apical levels; however, no statistically significant difference was observed in the number of osteoclasts at the root level between the two groups (Figure 6).

### 3.2. Percentages of Root Resorption

At baseline, the highest percentages of resorption of the distobuccal and distopalatal roots were at the apical level (distobuccal root: 3.43 ± 2.94 and distopalatal root: 3.07 ± 2.94). On day 21, the percentage of resorption at both roots in the CO group was slightly higher than in the CI group at all levels. However, root resorption at the distopalatal root of the CO group was higher at the middle to apical level. The percentages of root resorption at the distobuccal root of the CO group and the distobuccal and distopalatal roots of the CI group were higher at the cervical to middle level. Both experiment groups had higher root resorption than the control group; however, no significant differences were found between the control, CO, and CI groups (Table 1).

## 4. Discussion

Selective corticotomy is induced bone damage associated with local surgical bone remodeling. The biological mediators generated by resident cells are an initial inflammatory phase and intense osteoclastic bone resorption, which presents as transient osteopenia. Osteoclastic bone resorption, which is a fundamental element of osteoclastogenesis and a participant in bone remodeling to enable tooth movement, generates physiological bone remodeling [20].

The findings of this study supported earlier research by showing the highest increase in the number of osteoclasts was during the early stages of tooth movement and gradually decreased in the second and third weeks. The highest increase during the first week after orthodontic tooth movement combined with corticotomy was related to the maximum catabolic activity [21,22]. However, some previous research demonstrated that corticotomy and orthodontic tooth movement raised the number of osteoclast cells to their maximal level in the second week before declining [23,24]. This longer period to reach the maximum number of osteoclast cells was likely due to different animal species, force magnitudes, and appliances used.

Since the corticotomy procedure is associated with moderate to severe pain in the first week, pain management should be taken into consideration to reduce discomfort [7]. Ibuprofen is a drug that is recommended to use in order to recover from dental surgery [25] since it has a consistent analgesic and anti-inflammatory action, which helps to control pain, and it is cost-effective [9,26]. Ibuprofen administration has been associated with a delay in orthodontic tooth movement [13,14,15] by a decrease in PGE_2_ secretion [12,13] and osteoclast numbers [14].

According to previous animal research, the rate of tooth movement during corticotomy-assisted orthodontic therapy with or without ibuprofen administration was not significantly different [16]. In this present study, no significant differences were observed in the number of osteoclasts between the CO and CI groups during any of the experimental periods. Furthermore, the corticotomy induced a more robust cell response than the anti-inflammatory pharmaceutical effect. Therefore, ibuprofen administration had no significant impact on the osteoclastic cell response due to corticotomy-facilitated orthodontic treatment.

Our study found that the control group experienced only 1 to 3% root resorption, while the CO group experienced root resorption of 2.3 to 5.1% in the distobuccal root and 1.3 to 3.5% in the distopalatal root (Table 1). In rats treated with corticotomy and light orthodontic force for 28 days, the distobuccal root and distopalatal root were also found to have root resorption rates that were approximately 12.54% and 7.95%, respectively [17]. The different characteristics of root resorption were the consequence of the various treatment time points. Previous studies showed that corticotomy with light force could accelerate tooth movement by rapidly eliminating hyalinization without increasing the amount of root resorption [23].

Although there were no statistically significant differences between the two corticotomy-treated groups, the CO group exhibited a higher percentage of root resorption than the CI group. This finding corresponds with a study on the effectiveness of ibuprofen in preventing root resorption during orthodontic tooth movement [15]. Nevertheless, based on our results, no statistically significant differences in root resorption were observed between the three groups, which indicated that corticotomy and ibuprofen had no significant influence on root resorption.

Corticotomy with ibuprofen administration during orthodontic tooth movement affected the biological response, as evidenced by a non-significantly lower number of osteoclasts compared with corticotomy alone in this study. This was likely related to the pharmacological effect of ibuprofen by inhibiting PGE_2_ levels [12,13]. Our investigation showed that low dosages of an anti-inflammatory medication had no significant influence on the number of osteoclasts and the degree of root resorption. Osteoclasts are an important component of the biological response to bone-tissue injury that is mediated by various growth factors, chemokines, and cytokines. Ibuprofen may therefore be advantageous for patients who undergo orthodontic therapy that is greatly facilitated by corticotomy. As rats and humans have different tissue compositions and physiological responses, there are several factors that are related to tooth movement. In order to better understand the entire mechanism of the inflammatory response to corticotomy-facilitated orthodontic tooth movement, further immunochemistry investigation and human studies are recommended.

## 5. Conclusions

The corticotomy-facilitated orthodontic tooth movement with or without ibuprofen administration demonstrated significantly increased numbers of osteoclasts compared to the normal bone of the control group. However, no significant effects on the number of osteoclasts were observed between the corticotomy and corticotomy with ibuprofen intake groups. There were no statistically significant differences between the roots of the control and the corticotomy groups with or without ibuprofen intake. Furthermore, the administration of ibuprofen in orthodontic treatment combined with the corticotomy procedure had no significant effect on root resorption.

## Figures and Tables

**Table 1:** Comparison of percentages of root resorption between the control, CO, and CI groups.

		Root Resorption (%)			*p*-Value
		Control	CO	CI	Inter-Group
DB root	Cervical	1.78 (1.32) ^NS^	5.08 (2.94) ^NS^	2.30 (2.58) ^NS^	0.065
	Middle	1.78 (2.00) ^NS^	4.54 (3.27) ^NS^	3.48 (2.45) ^NS^	0.219
	Apical	3.43 (2.94) ^NS^	2.73 (2.36) ^NS^	1.32 (1.66) ^NS^	0.323
	*p*-value intra-group	0.350	0.361	0.286	
DP root	Cervical	0.94 (0.56) ^NS^	2.27 (1.16) ^NS^	2.13 (1.60) ^NS^	0.137
	Middle	1.23 (1.02) ^NS^	3.45 (4.04) ^NS^	3.31 (2.58) ^NS^	0.341
	Apical	3.07 (2.94) ^NS^	3.10 (2.61) ^NS^	1.50 (2.04) ^NS^	0.480
	*p*-value intra-group	0.091	0.700	0.345	

Values are presented as mean and standard deviation. ^NS^ = No significant differences, (One-way ANOVA, *p* < 0.05). DB = distobuccal, DP = distopalatal.

## Data Availability

Not applicable.
